# Supplementary material for: MITF activity is regulated by a direct interaction with RAF proteins in melanoma cells
Source: Commun Biol. 2022 Jan 28;5:101. doi: 10.1038/s42003-022-03049-w (PMC8799692; doi:10.1038/s42003-022-03049-w)

Supplementary Materials for

MITF activity is regulated by a direct interaction with RAF proteins in melanoma cells

Charlène Estrada[1,2,3,4,5], Liliana Mirabal-Ortega[1,2,3,4,5,6], Laurence Méry[1,2,3,4,5,6], Florent Dingli[7], Laetitia Besse[8,9], Cedric Messaoudi[8,9], Damarys Loew[7], Celio Pouponnot[1,2,3,4,5,6], Corine Bertolotto[10], Alain Eychène[1,2,3,4,5,6] and Sabine Druillennec[1,2,3,4,5,6*]

[1] Institut Curie, Centre de Recherche, Orsay F-91405, France

[2] INSERM U1021, Centre Universitaire, Orsay F-91405, France

[3] CNRS UMR 3347, Centre Universitaire, Orsay F-91405, France

[4] Université Paris-Saclay, Orsay F-91405, France

[5] PSL Research University, Paris F-75006, France

[6] Equipe Labellisée Ligue Nationale Contre le Cancer, Orsay F-91405, France

[7] Institut Curie, PSL Research University, Centre de Recherche, Laboratoire de Spectrométrie de Masse Protéomique, 26 rue d'Ulm, Paris 75248 Cedex 05, France

[8] Institut Curie, PSL Research University, CNRS UMS 2016, F-91401 Orsay, France

[9] Université Paris-Saclay, INSERM US43, F-91401 Orsay, France

[10] Université Côte d'Azur, INSERM U1065, Centre Méditerranéen de Médecine Moléculaire (C3M), Nice, France.

Running title: Control of MITF activity by RAF binding

[*] Correspondence:

Dr. Sabine Druillennec e-mail: sabine.druillennec-rodiere@curie.fr

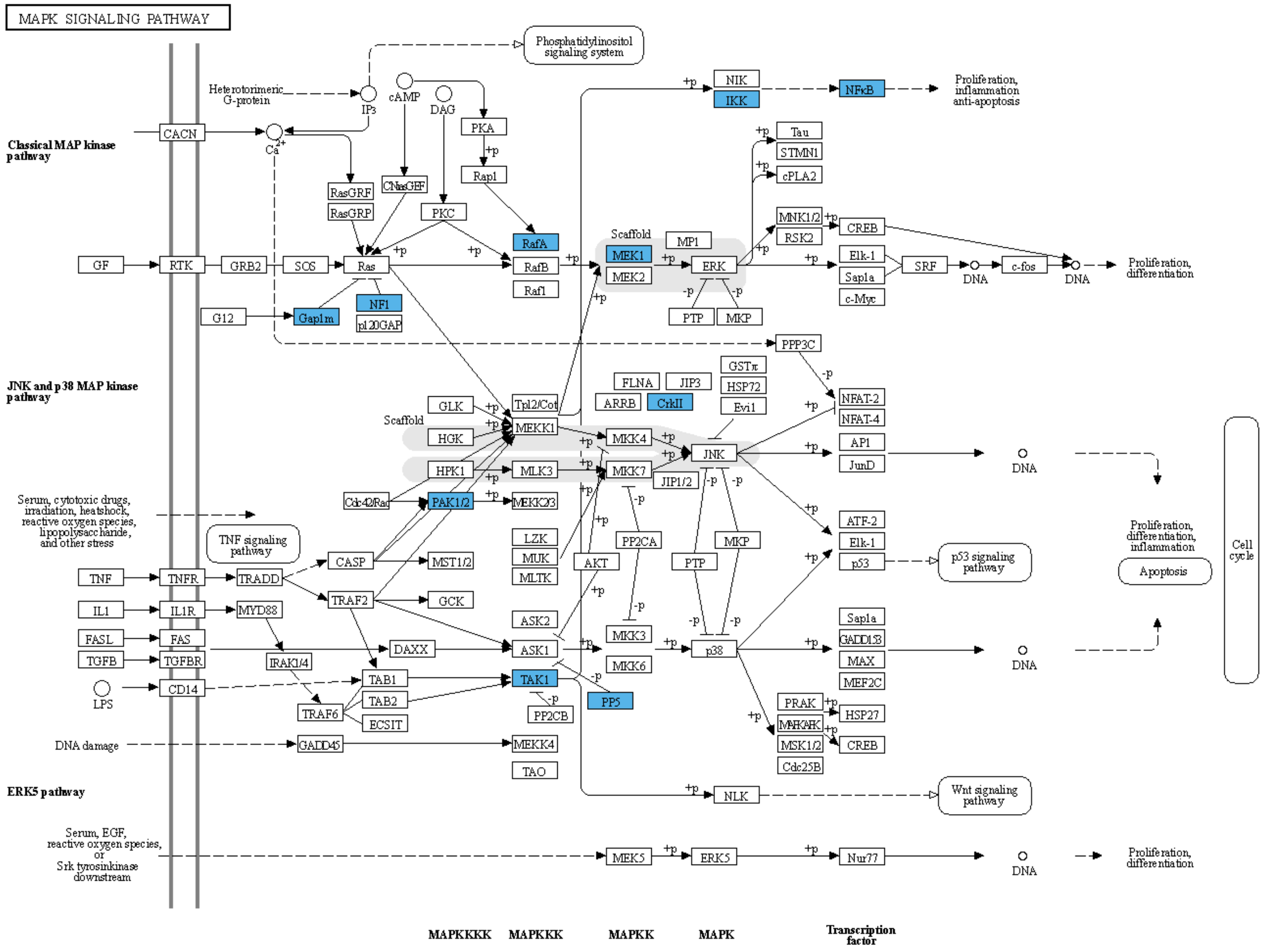

Supplementary Figure 1 **KEGG pathway analysis of a subset of 431 ARAF interactors.**

KEGG analysis of MAPK signaling pathway on a subset of 431 ARAF interactors found enriched in ARAF only cells by using the pathview R package. Several interactors of ARAF are highlighted in blue.

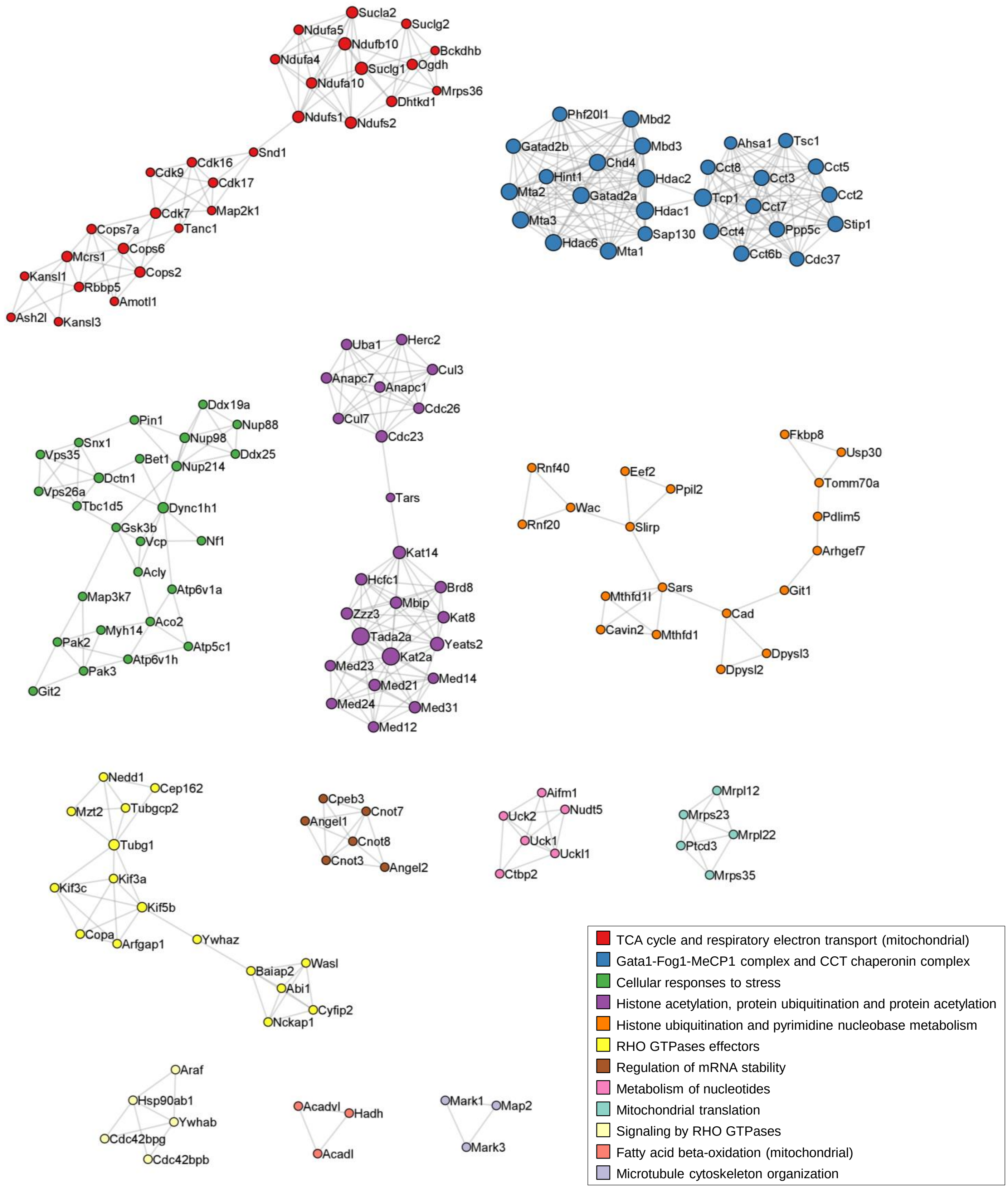

Supplementary Figure 2 Protein-protein network analysis of a subset of 431 ARAF interactors enriched in ARAF only cells.

Protein-protein interaction enrichment analysis, performed with Metascape and visualized with Cytoscape, using a list of 431 ARAF interactors. The network contains the subset of proteins (175 in total), represented by the nodes, that physically interact with at least one other member in the list. Node size indicates the connectivity degree. Interactions between proteins are represented by grey edges (510 edges in total). All the networks gathering between 3 and 500 proteins are used and are the output of molecular complex detection algorithm used by Metascape to identify densely connected network components. Functional terms, labelled by the color-code, provide a summary of network functional description.

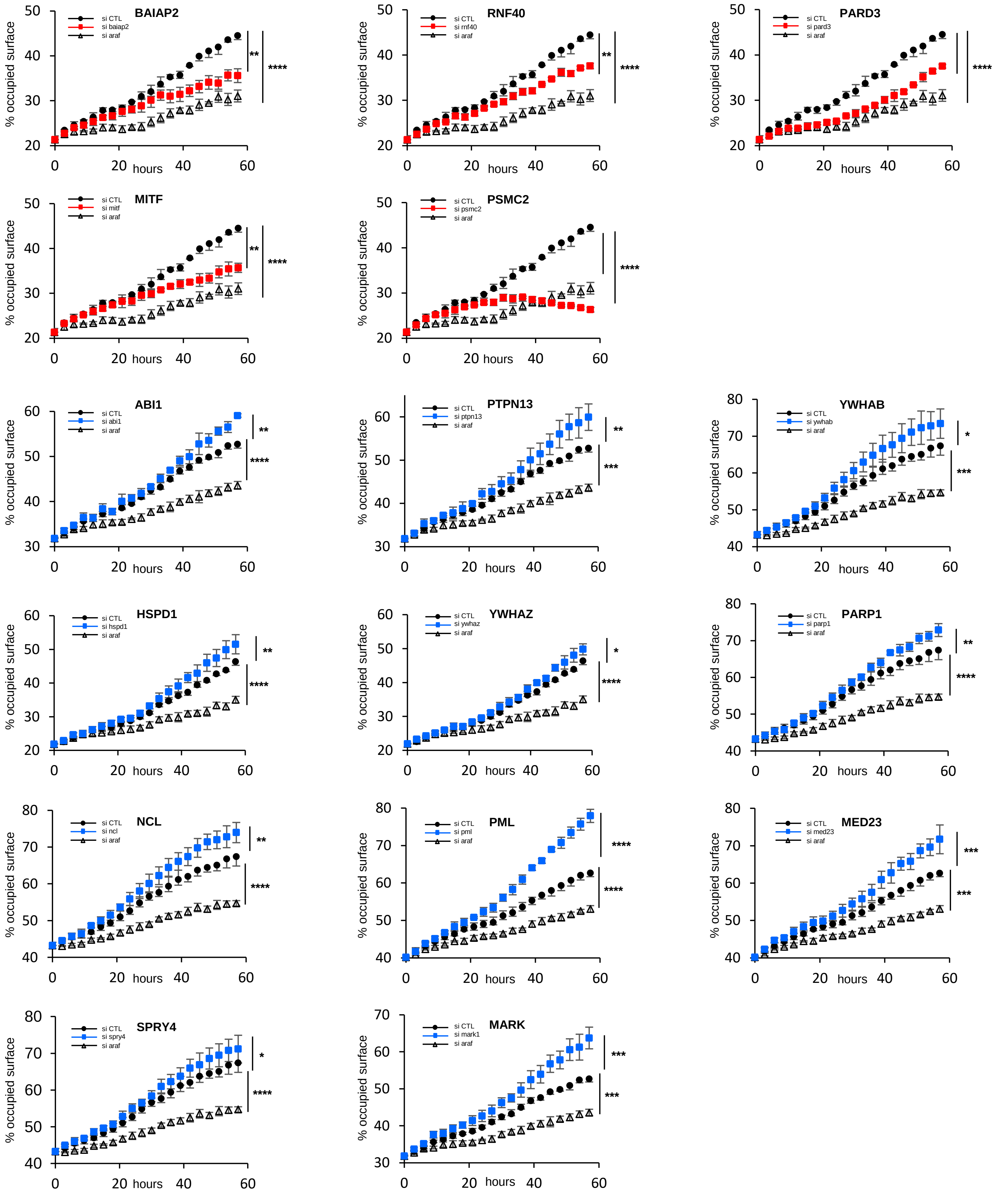

Supplementary Figure 3 Functional siRNA-based screen for a subset of 16 interactors identified by proteomics. Proliferation followed by IncuCyte® of ARAF-only cells after transfection with a pool of siRNA against a selected interactor (squares in red or blue), control siRNA (siCTL, black circle) or siRNA pool against ARAF (siARAF, open black triangle). Knock-down of the selected interactors induced either anti-proliferative (for 5 interactors highlighted in red, top panels) or pro-proliferative effect (for 11 interactors highlighted in blue, bottom panels). Data are the mean with standard deviations of three or four replicates. * p-value<0.05, ** p-value<0.01, *** p-value<0.001, **** p-value<0.0001 compared by a two-way ANOVA with Dunnett's multiple comparisons test.

Supplementary Fig. 4 Estrada et al.

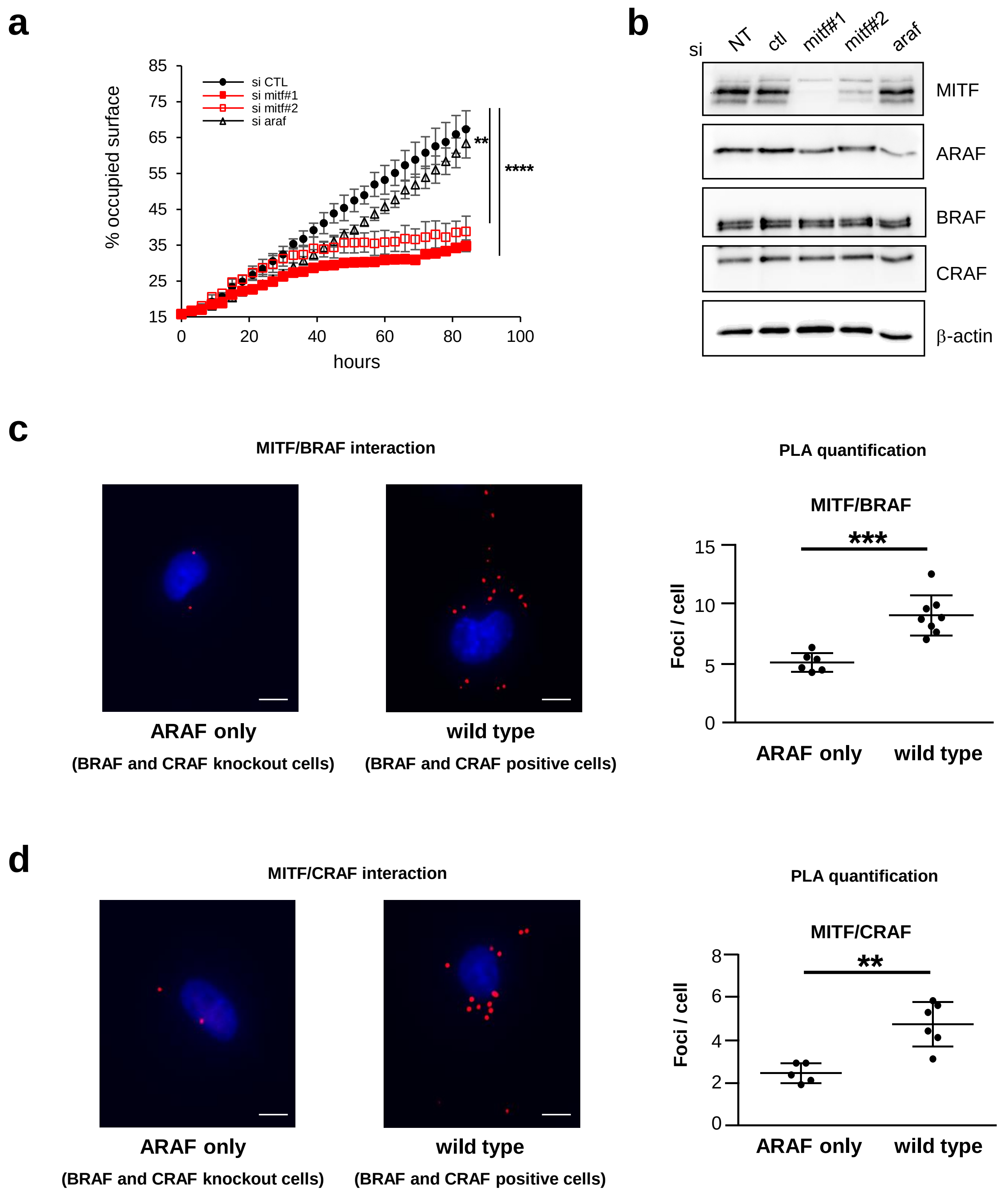

Supplementary Fig. 4 Endogenous BRAF/MITF and CRAF/MITF complexes in murine melanoma cells.

a Proliferation of NRAS-mutated murine melanoma cells, named wild type cells, after transfection with a control siRNA (siCTL, black circles), individual siRNA against MITF (siMITF #1 or siMITF #2, in red squares) or siRNA pool against ARAF (siARAF, open black triangles). Wild type cells express endogenous ARAF, BRAF and CRAF. Data are the mean with standard deviations of four replicates ($n = 4$). ** p-value<0.01, **** p-value<0.0001 compared by a two-way ANOVA with Dunnett's multiple comparisons test. **b** Expression of MITF, ARAF, BRAF and CRAF by western blot analysis in wild type cells non-transfected (NT) or transfected with either siCTL, siMITF or siARAF. β-actin is used as a loading control. **c, d** Identification of endogenous BRAF/MITF (**c**) and CRAF/MITF (**d**) complexes by Proximity Ligation Assay. Complexes were visualized as red dots by using a fluorescent microscope in wild type cells. Cell nuclei were stained with DAPI. ARAF-only cells which are deficient for BRAF and CRAF were used as a control. Scatter plots represent the average number of dots per cell (at least 234 nuclei were observed) of five to eight microscopic fields. Representative pictures from three independent experiments are shown. Means with standard deviations are shown. *** p-value=0.0002, ** p-value<0.0014 compared by unpaired t-test. Scale bar: 100 µm.

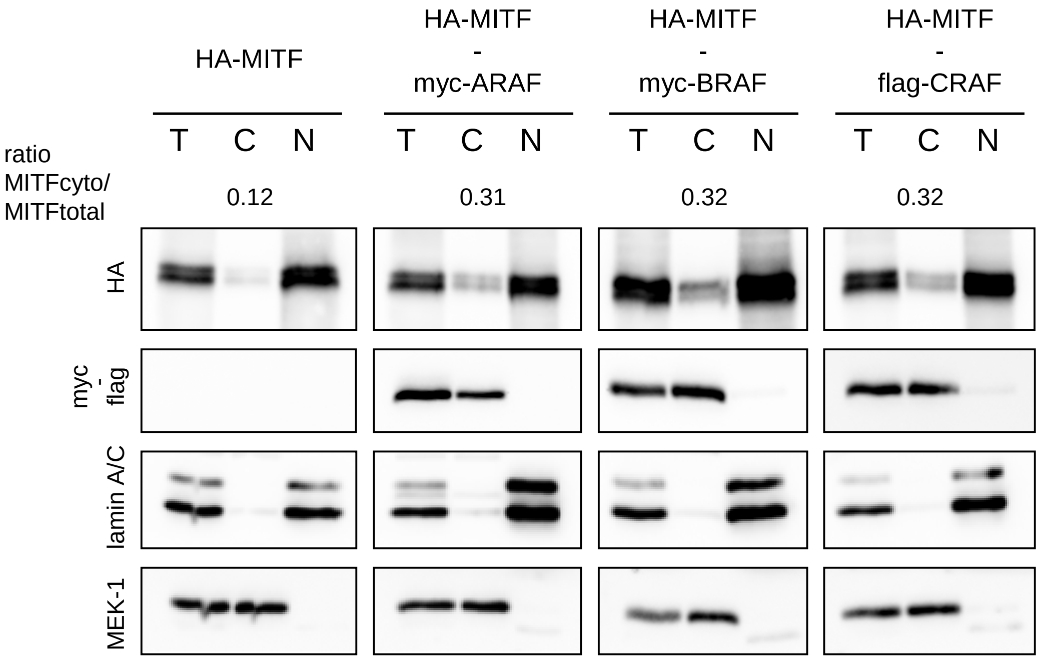

Supplementary Figure 5 MITF localisation by subcellular fractionation.

HEK293T cells were cotransfected with HA-MITF and myc-ARAF, myc-BRAF, flag-CRAF or control vector. After fractionation, MITF and RAF protein localisation was determined by western blot by using anti-HA and anti-myc or flag antibodies, respectively. Subcellular fractions are abbreviated as T for total cell extract, C for cytoplasmic fraction and N for nuclear fraction. Lamin A/C or MEK1 immunostaining was used as nuclear or cytoplasmic marker, respectively. The quantification of cytoplasmic MITF compared to total MITF (MITF cyto/MITF total) was obtained by dividing the ratio of cytoplasmic MITF over cytoplasmic MEK-1 by the ratio of total MITF over total MEK-1. This figure is representative of three independent experiments.

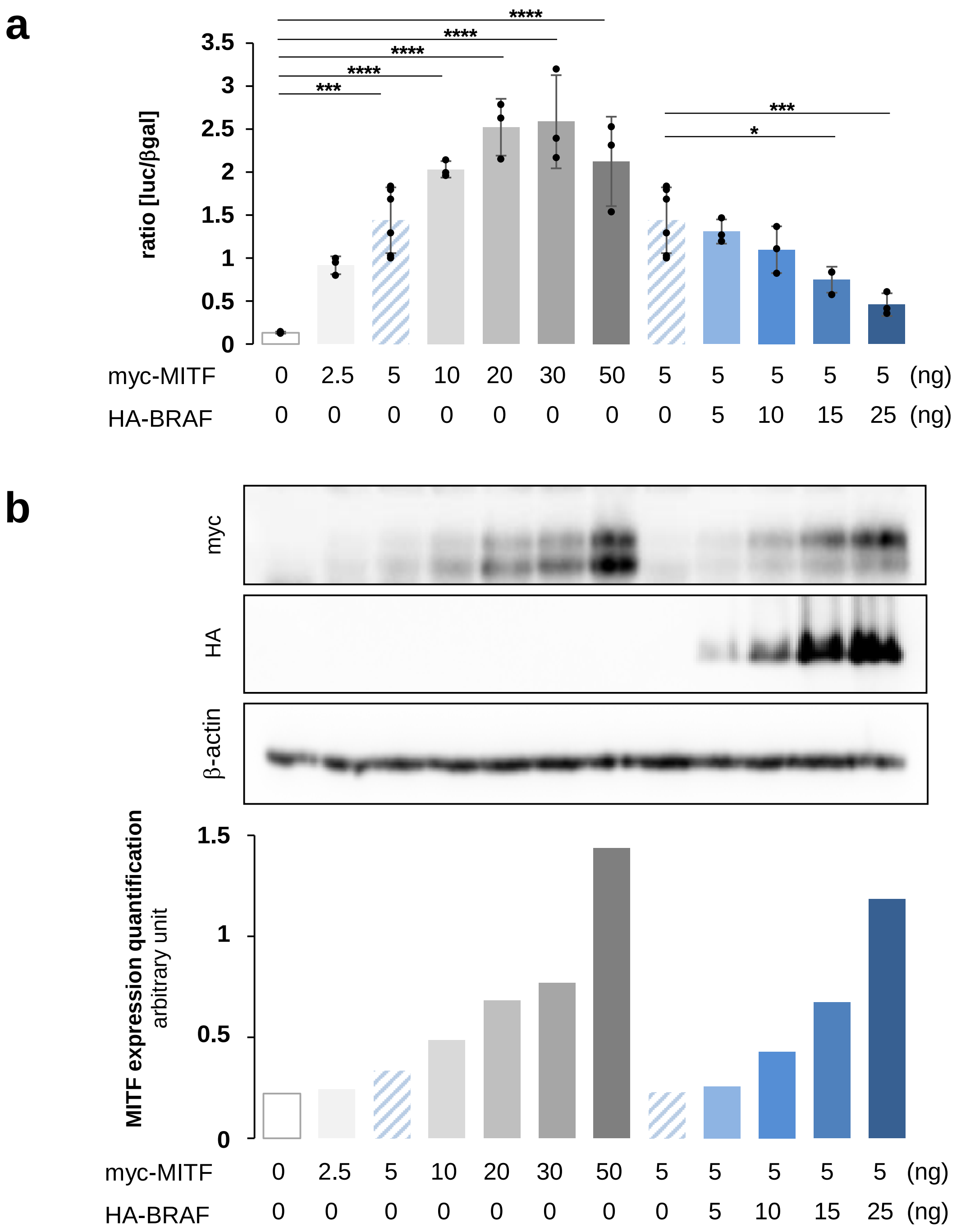

Supplementary Fig. 6 MITF expression in luciferase assays.

HEK293T cells were cotransfected with MITF plasmid and either empty constructs or BRAF plasmid at the indicated doses in the presence of a *TYR*-Luc luciferase reporter and a control β-galactosidase reporter. **a** The ratio of luciferase to β-galactosidase activities is shown as the mean with standard deviations of three replicates ($n = 3$). One-way ANOVA test was performed to compare all conditions and Dunnett's tests for the multiple comparisons to the no MITF condition (* p-value <0.05; *** p-value<0.001; **** p-value<0.0001). **b** MITF and BRAF protein expression was measured by western blot with anti-myc or anti-HA antibodies, respectively. β-actin is used as a loading control. MITF expression was quantified by dividing myc signal by β-actin signal. Of note, the pcdna3-MITF plasmid drives MITF expression thanks to a CMV promoter that responds to the MAPK pathway activation, thus explaining the increase in MITF protein level following BRAF expression. A positive correlation between MITF activity and expression levels is observed with escalating amounts of MITF in absence of BRAF when a negative correlation is found if increasing amounts of BRAF are used at a constant dose of MITF.

Supplementary Fig. 7. Estrada et al.
Raw images related to the indicated figures (continued on next page)

Source data for Figure 1d

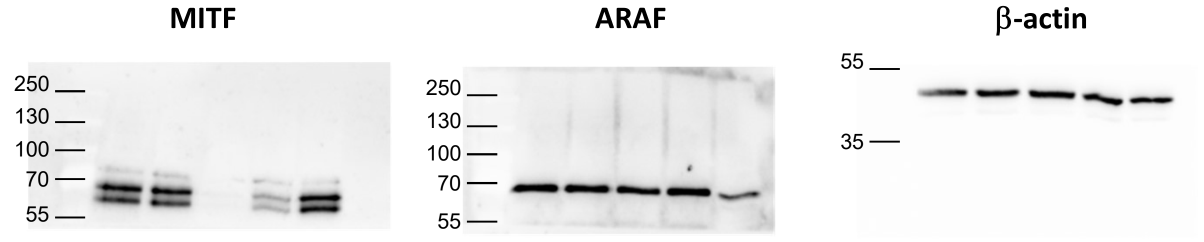

Source data for Figure 2a

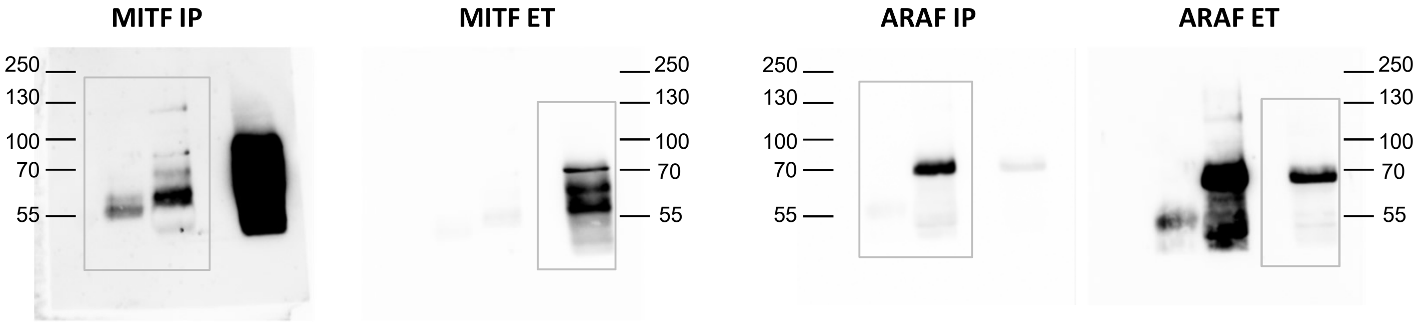

Source data for Figure 2c

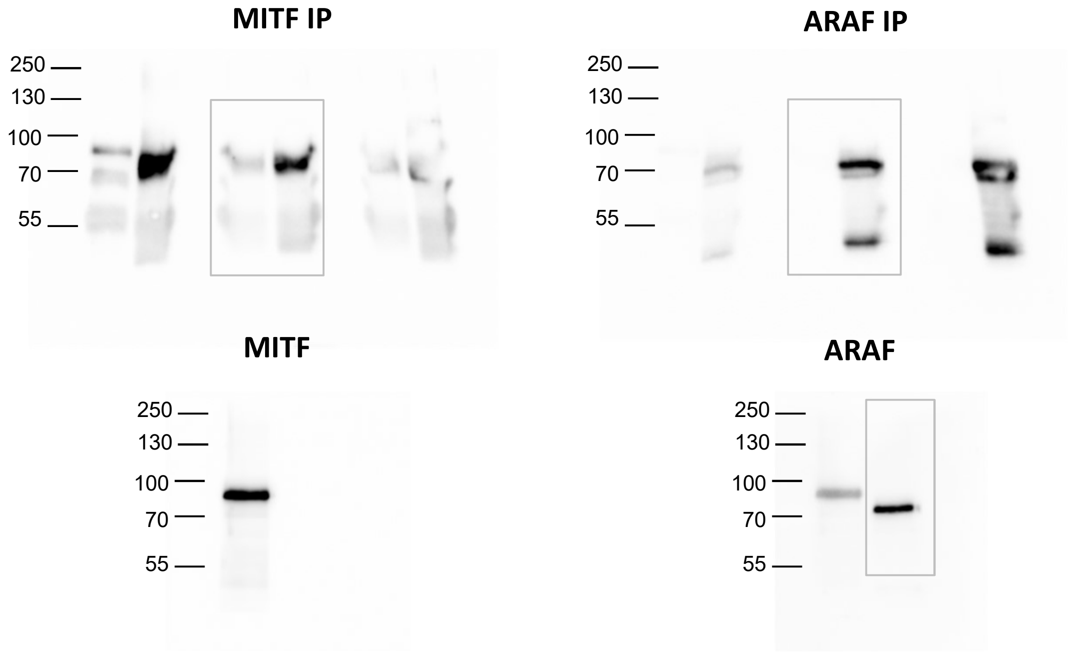

Supplementary Fig. 7. Estrada et al.
Raw images related to the indicated figures (continued on next page)

Source data for Figure 3a

MITF IP **MITF ET** **HA IP** **HA ET**

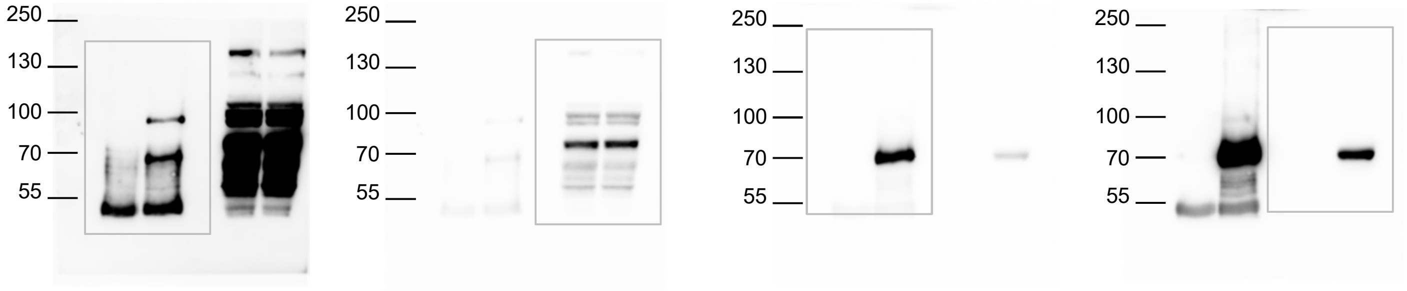

Source data for Figure 3b

MITF IP **MITF ET** **HA IP** **HA ET**

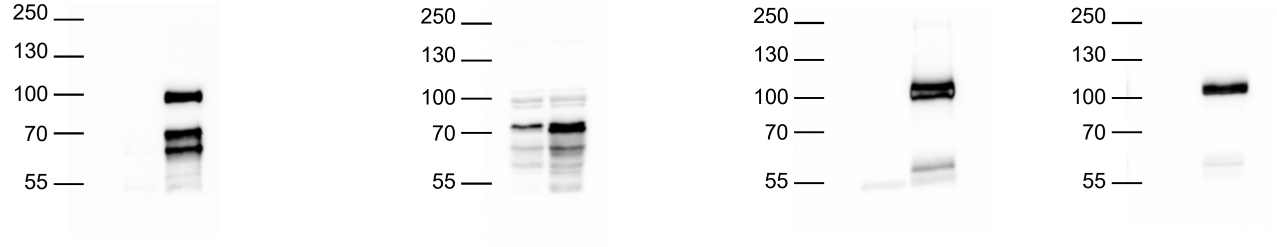

Source data for Figure 3c

MITF IP **MITF ET** **HA IP** **HA ET**

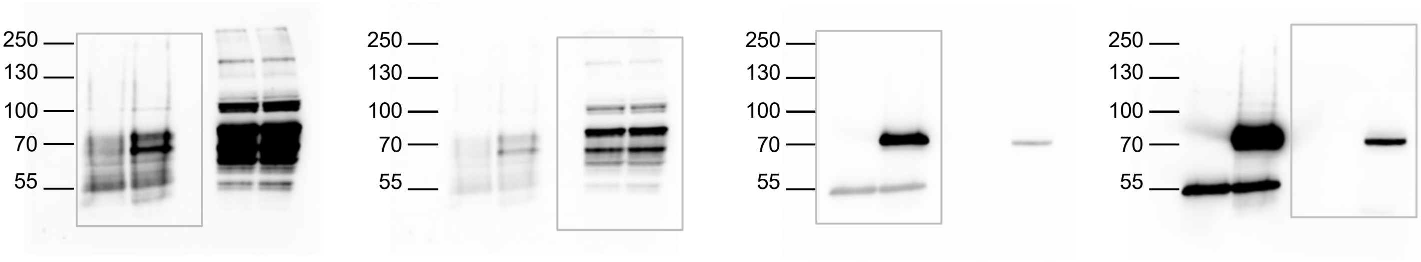

Source data Figure 3d

HA IP **HA ET** **myc IP** **myc ET**

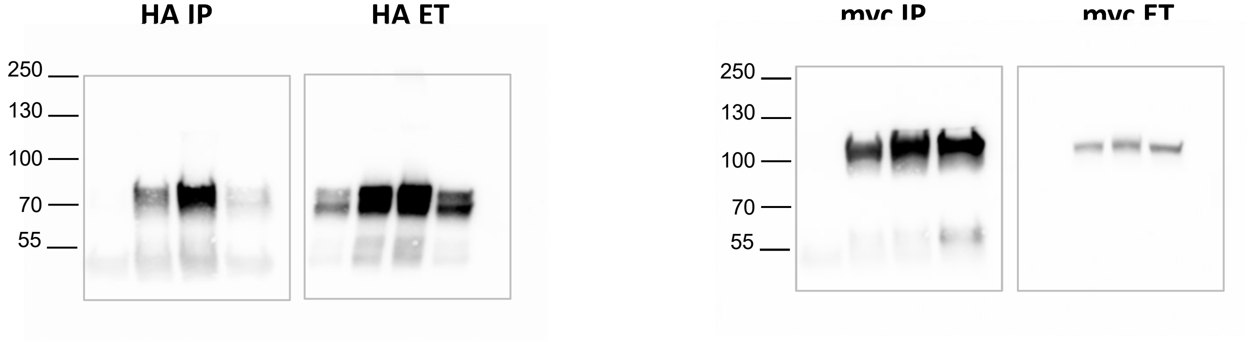

Supplementary Fig. 7. Estrada et al.
Raw images related to the indicated figures (continued on next page)

Source data for Figure 3e

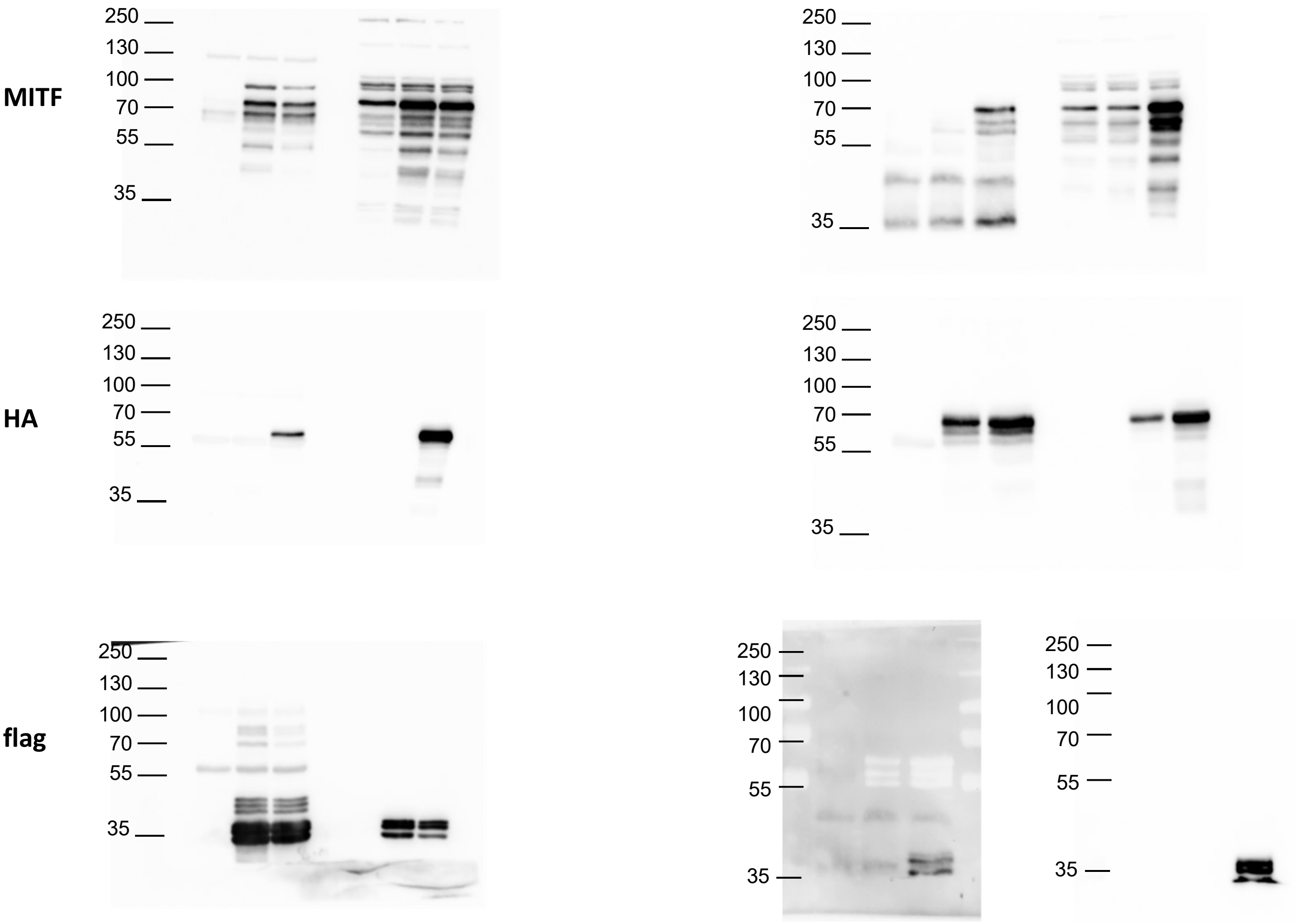

Source data for Figure 4a

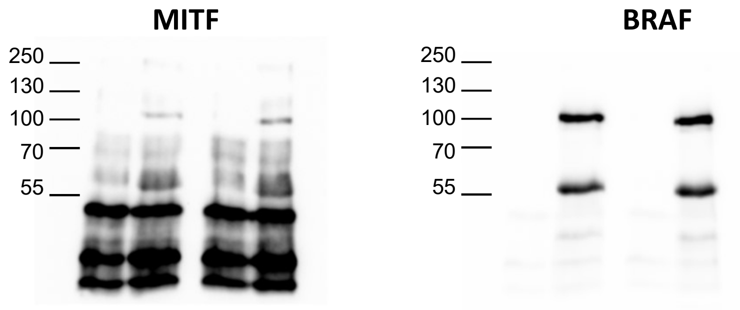

Source data for Figure 4b

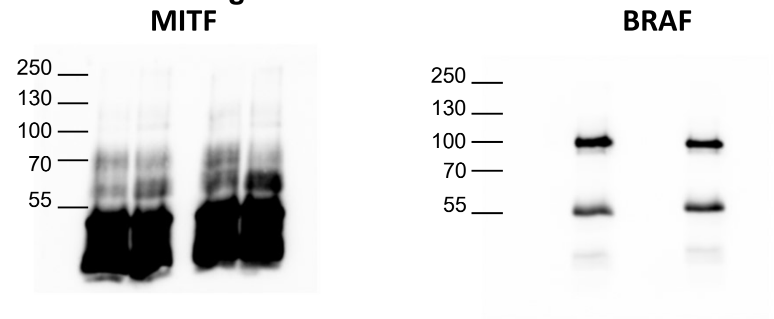

Supplementary Fig. 7. Estrada et al.
Raw images related to the indicated figures (continued on next page)

Source data for Figure 4c

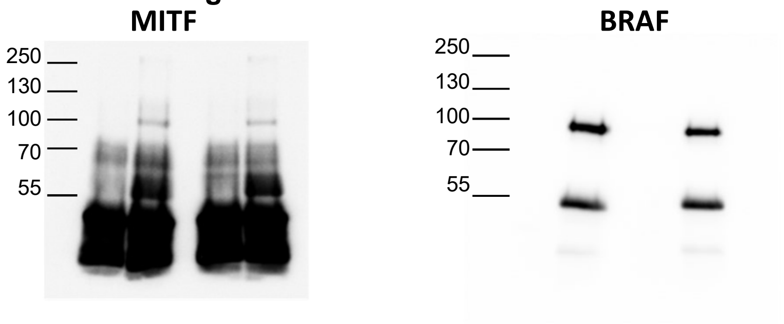

Source data for Figure 4d

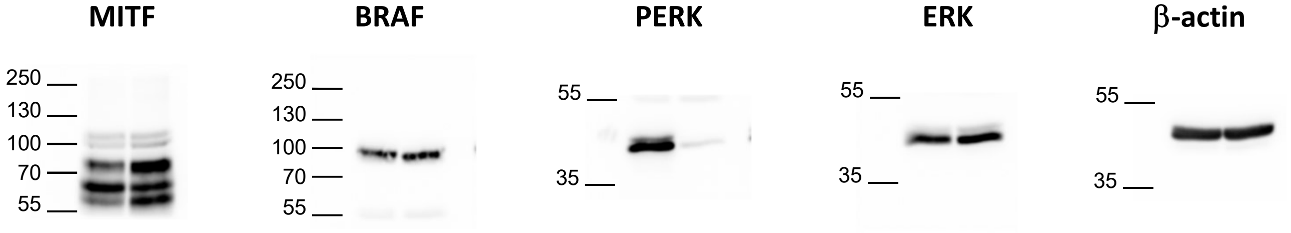

Source data for Figure 4e

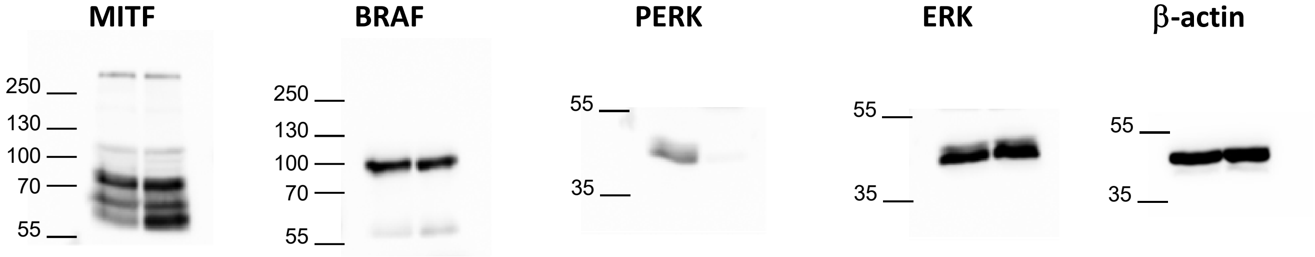

Source data for Figure 4f

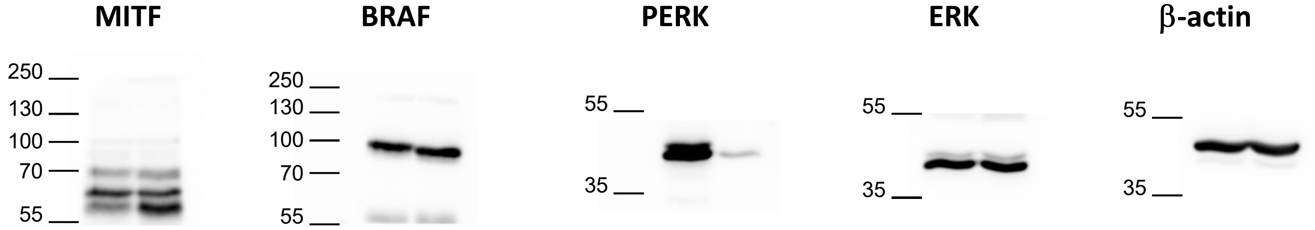

Source data for Supplementary Fig. 4b

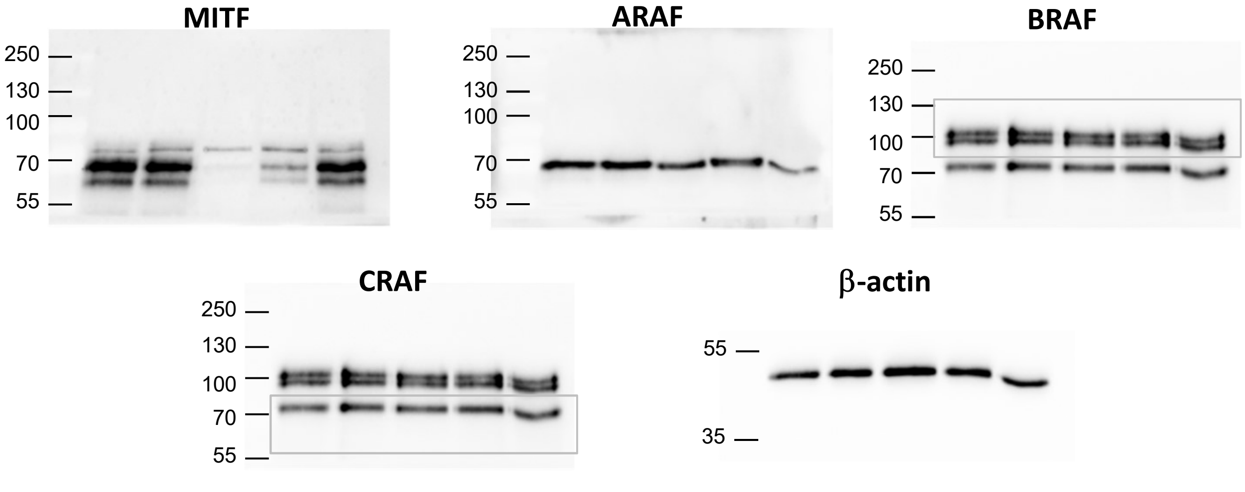

Supplementary Fig. 7. Estrada et al.
Raw images related to the indicated figures

Source data for Supplementary Fig. 5

HA

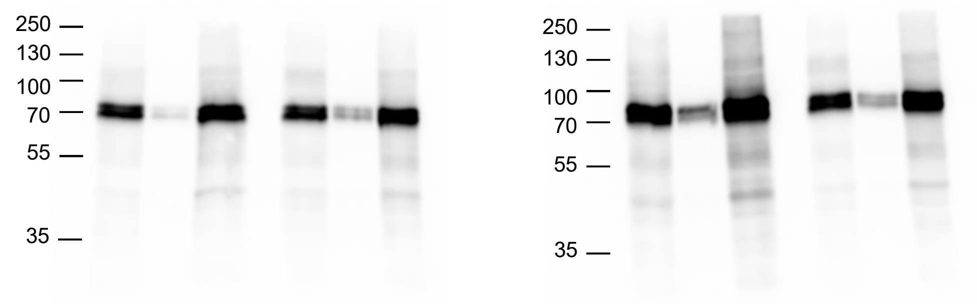

myc

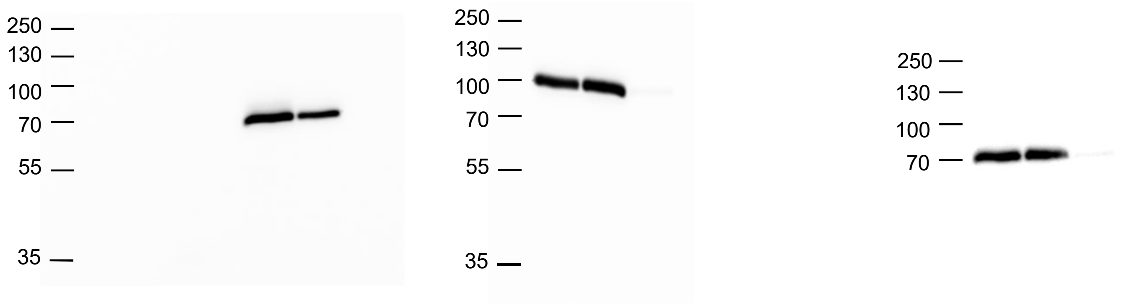

flag

laminA/C

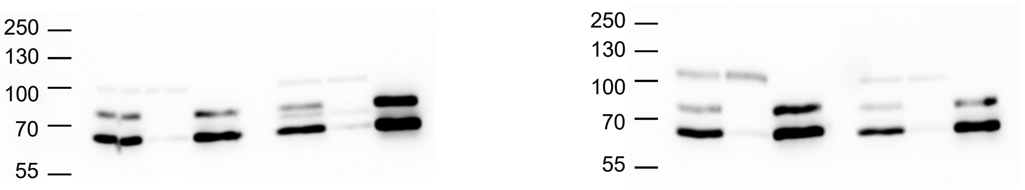

MEK-1

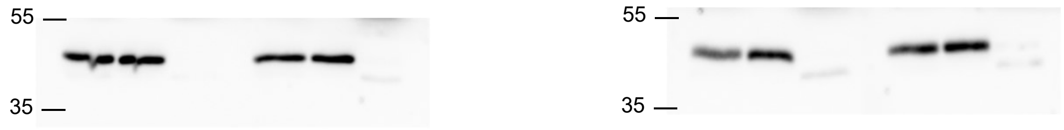

Source data for Supplementary Fig. 6b

myc

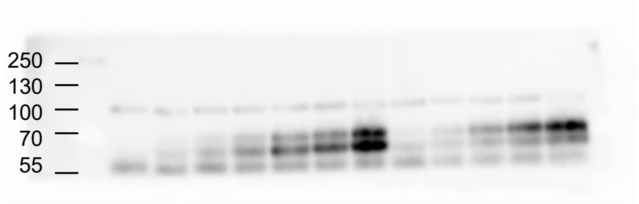

HA

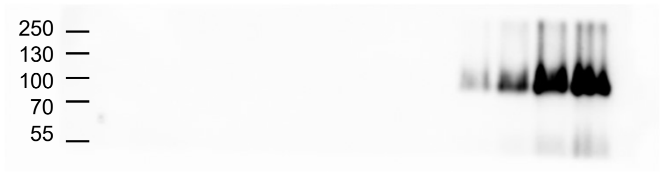

β-actin

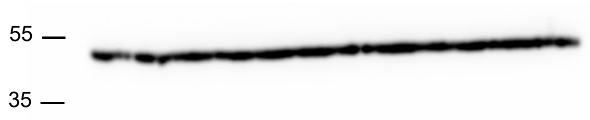

Supplement: Supplementary file 2 — Supplementary Information [file 42003_2022_3049_MOESM2_ESM.pdf]
