# Supplementary material for: MITF activity is regulated by a direct interaction with RAF proteins in melanoma cells
Source: Commun Biol. 2022 Jan 28;5:101. doi: 10.1038/s42003-022-03049-w (PMC8799692; doi:10.1038/s42003-022-03049-w)
Supplement: Supplementary file 3 — Description of Additional Supplementary Files [file 42003_2022_3049_MOESM3_ESM.pdf]

Description of Additional Supplementary Files

File name: Supplementary Data 1

Description: List of the 2700 proteins identified by mass spectrometry.

File name: Supplementary Data 2

Description: List of 431 ARAF interactors used for bioinformatics analysis. A list of 431 ARAF interactors specifically enriched in ARAF only cells, selected as followed: i) infinite ratio or ii) ratio > 2, p-value < 0.001, number of peptide used ≥ 9, was given as input ("Input table" tab). Final input list, with corresponding Entrez gene ID, is shown in "Annotation" tab. Enrichment results ("Enrichment" tab) present the top-20 clusters ("groupID" column) with their representative enriched terms that are grouped based on their membership similarities. The most statistically significant term within a cluster is chosen to represent the cluster. Term can be originated from different ontology sources (GO Biological Processes, KEGG Pathway, Reactome Gene Sets, CORUM or WikiPathways) mentioned in "Category" column.

File name: Supplementary Data 3

Description: List of the 99 putative ARAF partners selected for the siRNA-based functional screen. 69 were enriched in ARAF-only cells with the following parameters number of peptides≥9, ratio>2 and adjusted p-value<0.001, 15 exclusively identified in ARAF-only cells, and 15 are commonly found with the ARAF interactome published by Zhang *et al.* [33]. Proteins inducing an anti- or pro-proliferative effect after silencing by siRNA are highlighted in blue and red, respectively in the table and indicated in Fig. 1a.

File name: Supplementary Data 4

Description: Source data for the graphs and charts in the main figures.
