# Supplementary material for: MITF activity is regulated by a direct interaction with RAF proteins in melanoma cells
Source: Commun Biol. 2022 Jan 28;5:101. doi: 10.1038/s42003-022-03049-w (PMC8799692; doi:10.1038/s42003-022-03049-w)
Supplement: Supplementary file 8 — Reporting Summary [file 42003_2022_3049_MOESM8_ESM.pdf]

nature research

Corresponding author(s): manuscript COMMSBIO-21-0906

Last updated by author(s): Dec 9, 2021

Reporting Summary

Nature Research wishes to improve the reproducibility of the work that we publish. This form provides structure for consistency and transparency in reporting. For further information on Nature Research policies, see our Editorial Policies and the Editorial Policy Checklist.

Statistics

For all statistical analyses, confirm that the following items are present in the figure legend, table legend, main text, or Methods section.

n/a	Confirmed	
☐	☒	The exact sample size (*n*) for each experimental group/condition, given as a discrete number and unit of measurement
☐	☒	A statement on whether measurements were taken from distinct samples or whether the same sample was measured repeatedly
☐	☒	The statistical test(s) used AND whether they are one- or two-sided<br>*Only common tests should be described solely by name; describe more complex techniques in the Methods section.*
☒	☐	A description of all covariates tested
☐	☒	A description of any assumptions or corrections, such as tests of normality and adjustment for multiple comparisons
☐	☒	A full description of the statistical parameters including central tendency (e.g. means) or other basic estimates (e.g. regression coefficient) AND variation (e.g. standard deviation) or associated estimates of uncertainty (e.g. confidence intervals)
☐	☒	For null hypothesis testing, the test statistic (e.g. *F*, *t*, *r*) with confidence intervals, effect sizes, degrees of freedom and *P* value noted<br>*Give P values as exact values whenever suitable.*
☒	☐	For Bayesian analysis, information on the choice of priors and Markov chain Monte Carlo settings
☒	☐	For hierarchical and complex designs, identification of the appropriate level for tests and full reporting of outcomes
☒	☐	Estimates of effect sizes (e.g. Cohen's *d*, Pearson's *r*), indicating how they were calculated

Our web collection on statistics for biologists contains articles on many of the points above.

Software and code

Policy information about availability of computer code

Data collection	- For proteomics, Q Exactive HF-X, 2.9 SP0 Build 2923<br>- For western blot images acquisition : cooled-CDD camera Fusion FX Spectra, Vilber, Fusion FX7 Edge (v 18.04)<br>- For imaging (both Proximity Ligation Assay and immunofluoresecnce) : 3D/optigrid Leica fluorescent microscope, Leica MetaMorph (v 2.1.0)<br>- For luminescence data acquisition (luciferase assay) : TriStar LB942, MikroWin 2010 (v 5.14)<br>- For proliferation follow up : Incucyte S3 (v 2020B)
Data analysis	- For mass spectrometry peptide identification: Sequest–HT through proteome discoverer (version 2.0)<br>- For mass spectrometry relative quantification: MyProMS (v 3.5) = GitHub repository of myProMS https://github.com/bioinfo-pf-curie/myproms<br>- For KEGG pathway analysis : R packages ClusterProfiler (v 4.0.5) and Pathview (v1.32.0) used on R (4.1.1).<br>- For processs and pathway enrichment analysis : Metascape online tool (https://metascape.org)<br>- For statistical analysis of si-RNA based screen and luciferase assay : Graphpad Prism (v 9.1.0)<br>- For PLA couting and Western blotting quantification : ImageJ (v 1.53c)

For manuscripts utilizing custom algorithms or software that are central to the research but not yet described in published literature, software must be made available to editors and reviewers. We strongly encourage code deposition in a community repository (e.g. GitHub). See the Nature Research guidelines for submitting code & software for further information.

Data

Policy information about availability of data

All manuscripts must include a data availability statement. This statement should provide the following information, where applicable:

- Accession codes, unique identifiers, or web links for publicly available datasets
- A list of figures that have associated raw data
- A description of any restrictions on data availability

The proteomic datasets produced in this study are available in the PRIDE database.
Data that support findings of the present study are available upon reasonable request from the corresponding author.

Field-specific reporting

Please select the one below that is the best fit for your research. If you are not sure, read the appropriate sections before making your selection.

[x] Life sciences   [ ] Behavioural & social sciences   [ ] Ecological, evolutionary & environmental sciences

For a reference copy of the document with all sections, see nature.com/documents/nr-reporting-summary-flat.pdf

Life sciences study design

All studies must disclose on these points even when the disclosure is negative.

Sample size	Sample sizes were chosen on the basis of preliminary experiments.
Data exclusions	No data were excluded from the analyses.
Replication	All attempts at replication were successful. When appropriate, we performed at least three independent experiments.
Randomization	not applicable
Blinding	Analysis of PLA and immunofluorescence experiments was blindly performed.

Reporting for specific materials, systems and methods

We require information from authors about some types of materials, experimental systems and methods used in many studies. Here, indicate whether each material, system or method listed is relevant to your study. If you are not sure if a list item applies to your research, read the appropriate section before selecting a response.

Materials & experimental systems

n/a	Involved in the study	
[ ]	[x]	Antibodies
[ ]	[x]	Eukaryotic cell lines
[x]	[ ]	Palaeontology and archaeology
[x]	[ ]	Animals and other organisms
[x]	[ ]	Human research participants
[x]	[ ]	Clinical data
[x]	[ ]	Dual use research of concern

Methods

n/a	Involved in the study	
[x]	[ ]	ChIP-seq
[x]	[ ]	Flow cytometry
[x]	[ ]	MRI-based neuroimaging

Antibodies

Antibodies used

For mass spectrometry analysis :
ARAF antibody (75804, Cell Signaling)

For coimmunoprecipitation experiments :
anti-HA (3F10, Roche), anti-myc (9E10, Santa Cruz), anti-Flag (M2, Sigma), anti-ARAF antibody (#4432, Cell Signaling)

For Western Blot experiments :
anti-MITF (HPA003259, Sigma), anti-HA (3F10, Roche), anti-myc (9E10, Santa Cruz), anti-flag (M2, Sigma), anti-ARAF (4432, Cell Signaling), anti-BRAF (sc5284, Santa Cruz), anti-CRAF (610151, BD Biosciences), anti-ERK (sc93, Santa Cruz), anti-pERK (M8159, Sigma), anti-laminA/C (10298-1-AP, Proteintech), anti-MEK1 (sc219, Santa Cruz), anti-β-actin (A1978, Sigma), HRP-conjugated antibodies (HAF008 and HAF007, R&D Systems)

For proximity ligation assay experiments :
ARAF (4432, Cell Signaling), BRAF (F7, Santa Cruz), CRAF (#610151, BD Biosciences), MITF (ab12039, abcam), MITF (HPA003259, Sigma)

For immunofluorescence experiments:
anti-myc (9E10, Santa Cruz), anti-ARAF (4432, Cell Signaling), anti-MITF (HPA003259, Sigma), anti-BRAF (F7, Santa Cruz), anti-CRAF (610151, BD Biosciences), anti-mouse Alexa Fluor 594 (A21203), anti-rabbit Alexa Fluor 594 (A11037), anti-mouse Alexa Fluor 488 (A21202), anti-rabbit Alexa Fluor 488 (A11034)

Validation	Antibodies were validated by the manufacturers and we used appropriate controls for each experiment.

Eukaryotic cell lines

Policy information about cell lines

Cell line source(s)	HEK 293T (Human embryonic kidney) were from ATCC. Murine cell derived from NRAS-mutated murine melanoma have been already published (Dorard et al, Nature Comm, 2017). Human melanoma cell lines (A375 and SK28 from Nicolas Dumaz, Saint Louis Hospital, France and MelR from Caroline Robert, Gustave Roussy Hospital, France).
Authentication	Cell lines are regularly authenticated by STR testing.
Mycoplasma contamination	Cell are regularly tested for mycoplasma and were negative for these experiments.
Commonly misidentified lines (See ICLAC register)	No commonly misidentified cell lines were used.
